# Silibinin, a natural flavonoid, modulates the early expression of chemoprevention biomarkers in a preclinical model of colon carcinogenesis

**DOI:** 10.3892/ijo.2012.1526

**Published:** 2012-06-25

**Authors:** HENRIETTE KAUNTZ, SOUAD BOUSSEROUEL, FRANCINE GOSSE, JACQUES MARESCAUX, FRANCIS RAUL

**Affiliations:** 1 Laboratory of Nutritional Cancer Prevention, EA 4438, Faculty of Medicine, IRCAD, University of Strasbourg, Strasbourg;; 2 IRCAD - EITS, Strasbourg, France

**Keywords:** colorectal cancer, aberrant crypt foci, inflammation, apoptosis

## Abstract

The flavonolignan silibinin, the major biologically active compound of the milk thistle (*Silybum marianum*), has been shown to possess anticancer properties in a variety of epithelial cancers. The present study investigated the potential of silibinin as a chemopreventive agent in colon carcinogenesis. The rat azoxymethane (AOM)-induced colon carcinogenesis model was used because of its molecular and clinical similarities to sporadic human colorectal cancer. One week after AOM injection (post-initiation), Wistar rats received daily intragastric feeding of 300 mg silibinin/kg body weight per day until their sacrifice after 7 weeks of treatment. Silibinin-treated rats exhibited a 2-fold reduction in the number of AOM-induced hyperproliferative crypts and aberrant crypt foci in the colon compared to AOM-injected control rats receiving the vehicle. Silibinin-induced apoptosis in the colon mucosal cells was demonstrated by flow cytometry after propodium iodide staining and by colorimetric measurement of caspase-3 activity. Mechanisms involved in silibinin-induced apoptosis included the downregulation of the anti-apoptotic protein Bcl-2 and upregulation of the pro-apoptotic protein Bax, inverting the Bcl-2/Bax ratio to <1. This modulation already takes place at the mRNA expression level as shown by real-time RT-PCR. Furthermore, silibinin treatment significantly (P<0.01) decreased the genetic expression of biomarkers of the inflammatory response such as IL1β, TNFα and their downstream target MMP7, all of them shown to be upregulated during colon carcinogenesis. The downregulation of MMP7 protein was confirmed by western blot analysis. The present findings show the ability of silibinin to shift the disturbed balance between cell renewal and cell death in colon carcinogenesis in rats previously injected with the carcinogen AOM. Silibinin administered via intragastric feeding exhibited potent pro-apoptotic, anti-inflammatory and multi-targeted effects at the molecular level. The effective reduction of preneoplastic lesions by silibinin supports its use as a natural agent for colon cancer chemoprevention.

## Introduction

Colorectal carcinogenesis offers a large window spanning decades from cancer initiation to diagnosis making this disease suitable for chemopreventive approaches (1). With the aid of reliable preclinical models it is possible to evaluate the impact of potential chemopreventive agents on the carcinogenic process. These preclinical models include the induction of colon carcinogenesis in rats by the administration of the chemical carcinogen azoxymethane (AOM). Many investigators have used the AOM model to study the effects of various chemopreventive agents on colon carcinogenesis (2–4). Studies in humans and in the rat AOM model have identified early preneoplastic hyperproliferative lesions forming aberrant crypts and aberrant crypt foci (ACF) as intermediate biomarkers of the carcinogenic process (5–7). ACF have also been used as surrogate biomarkers to screen numerous potential chemopreventive agents (8).

The flavonolignan silibinin constitutes the major biologically active part of the extract silymarin isolated from the milk thistle plant (*Silybum marianum*) (9). Milk thistle extract has already been used as a hepatoprotective substance for more than 2,000 years and is known to be non-toxic. During the last decade, numerous studies showed that its main component, silibinin, exhibits anticancer and chemopreventive properties against various epithelial cancers such as skin (10), prostate (11), lung (12) and colorectal (13–16) cancer in different *in vitro* and *in vivo* models. Despite the numerous studies showing the chemopreventive potential of silibinin in different cancer models, its efficacy against colorectal cancer development in animal models remains less understood.

We previously selected a panel of gene and protein biomarkers involved in the inflammatory response and in the apoptotic pathway and shown to exhibit significant changes occurring early during the post-initiation stage in the colon mucosal cells after AOM injection (17). These data support the importance of the preclinical rat AOM model in the screening for new drugs designed for preventive and/or therapeutic activity against colorectal cancer. Here, we extended these investigations in order to determine the effects of dietary feeding of silibinin on the development of AOM-induced ACF formation, and on the expression of several gene and protein biomarkers involved in the inflammatory and apoptotic responses in the early post-initiation phases of colon carcinogenesis.

## Materials and methods

### Animals and treatments

All animal experiments were performed in accordance with the institutional guidelines of the French Ethics Committee (authorization no. A67-480, French Ministry of Agriculture). Male Wistar rats (n=24), obtained from Charles River Laboratories (Les Oncins, France) and weighing 200–220 g, were housed under standardized conditions (22°C, 60% relative humidity, 12 h light/12 h dark cycle, 20 air changes/h) and fed a standard diet with free access to drinking water. Sixteen rats received intraperitoneal injections of azoxymethane (AOM) (Sigma-Aldrich, Saint-Quentin Fallavier, France), at a concentration of 15 mg/kg body weight, once a week for 2 weeks. One week after the last injection of AOM (post-initiation), rats were randomly separated into two groups. One group (n=8) received every day silibinin via intragastric intubation [300 mg/kg body weight dissolved in 0.5% carboxymethyl cellulose (CMC)], while the AOM-control group (n=8) received every day the excipient (0.5% CMC). A third group of rats (n=8), injected with 0.9% NaCl (saline) once a week for 2 weeks, was used as reference and received the excipient (0.5% CMC) via intragastric intubation. All animals were sacrificed 7 weeks after AOM or saline injection.

### Assessment of aberrant crypts in the colon

The determination of aberrant hyperproliferative crypts was performed on a segment of 6 cm in length, corresponding to the distal part of the colon. The segment was washed with physiological saline, cut open, pinned out flat and fixed in 10% buffered formalin. The colon was stained with 0.2% methylene blue for 5 min, rinsed in Krebs-Ringer buffer, placed onto a glass slide and examined microscopically using a low-power objective (×5) to assess hyperproliferative crypts and aberrant crypt foci (ACF). The criteria for the identification of hyperproliferative aberrant crypts were: i) increased size, ii) thicker epithelial cell lining and iii) increased pericryptal zone relative to normal crypts. Mucosal samples of the distal colon of NaCl-injected rats, AOM-injected rats and silibinin-treated AOM-injected rats were scraped off with a glass slide and were immediately frozen in liquid nitrogen (for flow cytometry analysis mucosal cells were processed immediately).

### Flow cytometry analysis of sub-G0/G1 cell population

The sub-G0/G1 cell population (hypodiploïd cells, dying and dead cells) was analyzed by labeling cells with propidium iodide (18). In brief, the mucosa was removed from the underlying tissue by scraping with a glass slide and incubated in phosphate-buffered saline (PBS) containing 0.1 mg/ml collagenase (Sigma-Aldrich), with gentle stirring for 40 min at 37°C. Cell counts and viability were determined using trypan blue exclusion. Cells were washed twice with PBS and then fixed by the addition of 70% ethanol at −20°C for 30 min. The suspension was then centrifuged at 1,200 × g for 5 min at 4°C, washed twice with PBS and resuspendend in 200 *μ*l PBS containing 0.25 mg/ml RNase A and 0.1 mg/ml propidium iodide (Sigma-Aldrich). After incubation in the dark at 37°C for 30 min, the fluorescence of 10,000 cells/sample was analyzed by flow cytometry and histograms were analyzed by CellQuest software (FACScan; BD Biosciences, Erembodegem, Belgium).

### Measurement of caspase-3 activity

Caspase-3 activity was measured by using the colorimetric assay kit (MBL International Corporation, Nagoya, Japan), according to the manufacturer’s instructions. Mucosal cells were washed in ice-cold PBS; proteins were extracted and stored at −80°C. Cell lysates (20 *μ*l) were added to a buffer containing a p-nitroaniline (pNA)-conjugated substrate for caspase-3 (Ac-DEVD-pNA) to a total of 100 *μ*l reaction volume. Incubation was carried out at 37°C. The concentration of the released pNA was calculated from the absorbance values at 405 nm and the calibration curve of defined pNA solutions. Activities were expressed as fold-increase of the caspase activity measured in the mucosa of the NaCl-injected control rats.

### Real-time quantitative reverse-transcriptase polymerase chain reaction analysis

Total-RNA was isolated, using the RNeasy Plus Mini kit (Qiagen, Austin, TX, USA), from the scraped mucosa of NaCl-injected rats, AOM-injected rats and of silibinin-treated AOM-injected rats. The High Capacity cDNA Reverse Transcription kit (Applied Biosystems, Foster City, CA, USA) was used for cDNA synthesis as recommended by the supplier. RT-PCR was performed by using ABI TaqMan gene expression assays for MMP-7 (assay ID: Rn00563467), IL1β, TNFα (assay ID: Rn99999009, Rn99999017) and Bcl-2, Bax, (assay ID: Rn99999125, Rn02532082) according to the manufacturer’s instructions. All samples were run in triplicate in a 25 *μ*l reaction volume. Quantitative real-time RT-PCR was performed by using TaqMan Universal PCR master mix (Applied Biosystems) and the ABI Prism 7500 Sequence Detection System (Sequence detector; Applied Biosystems) in triplicate wells. The data were analyzed by a comparative threshold cycle (C_T_) method. C_T_ values were calculated using the 7500 SDS software (Applied Biosystems). The corresponding mRNA level from colonic mucosa of NaCl-injected control rats was used as an external reference. The level of β-actin mRNA (assay ID: Rn00667869) of each sample was used as an internal reference to normalize the data. The fold changes of each mRNA (mRNA relative expression) were expressed relative to the mean value of the corresponding mRNA found in the mucosa of the NaCl-injected control rats and was calculated using the 2^−ΔΔC_T_^ method (19).

### Western blot analysis of protein expression

Mucosal samples were homogenized in a RIPA lysis buffer composed of 150 mM NaCl, 50 mM Tris (pH 8.0), 5 mM EDTA, using a polytron homogenizer. After an ultracentrifugation for 30 min at 10,000 × g at 4°C, the protein content was measured by the Lowry method. Equal amounts of total protein were separated by a 15% SDS (sodium dodecyl sulfate)-polyacrylamide gel electrophoresis for 2 h and 30 min at 65 V. Then proteins were transferred to a nitrocellulose membrane (Bio-Rad Laboratories, Marnes-la-Coquette, France). The membrane was blocked with a solution, containing bovine serum albumin (BSA) 3%, Tween-20 0.1%, Tris-HCl 10 mM (pH 7.5) and 0.1% NaCl, for 1 h and was incubated overnight at 4°C with one of the following primary monoclonal antibodies: rabbit anti-rat MMP7 at 1:500 (Santa Cruz Biotechnology, Inc., Santa Cruz, CA, USA), rabbit anti-rat Bax at 1:500 (BD Biosciences), mouse anti-rat Bcl-2 at 1:100 (Calbiochem, Merck Biosciences, Darmstadt, Germany), or mouse anti-rat β-actin at 1:2,000 (Chemicon International, Inc., Hampshire, UK). The membranes were washed and incubated with 0.02 *μ*g/ml horseradish peroxidase (HRP)-conjugated goat anti-rabbit IgG (Calbiochem, Merck Biosciences) or with 0.02 *μ*g/ml HRP-conjugated goat anti-mouse IgG (Pierce, Perbio Science, Brebières, France) for 1 h and visualized using the Super Signal West Pico Chemiluminescent Substrate System (Pierce, Perbio Science).

### Statistical analysis

All data are presented as mean ± standard error (SE). Statistical differences were evaluated by one-way analysis of variance (ANOVA) and specific differences were identified using Student’s t-test.

## Results

### Silibinin inhibits the formation of preneoplastic lesions

**Figure 1:**
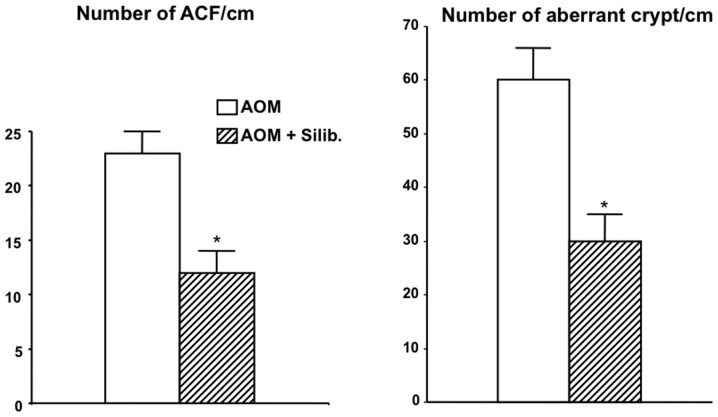
Number of aberrant crypt foci (ACF) and total number of aberrant crypts (per cm length) in the distal colon in AOM-injected control rats and in AOM-injected rats receiving silibinin. Data are presented as the mean ± SE (8 animals/group), ^*^P<0.01.

No significant variations in body weights were observed between the groups during the experimental period. The colon of NaCl-injected rats exhibited no preneoplastic lesions (i.e., aberrant crypts or ACF) in contrast to the colon of AOM-injected rats that always exhibited preneoplastic lesions. As illustrated in Fig. 1, animals treated with silibinin showed a 2-fold reduction in the number of hyperproliferative crypts compared to the AOM-injected control rats. Likewise, silibinin treatment reduced by 2-fold the amount of ACF in the colonic mucosa.

### Silibinin decreases inflammatory biomarkers

**Figure 2:**
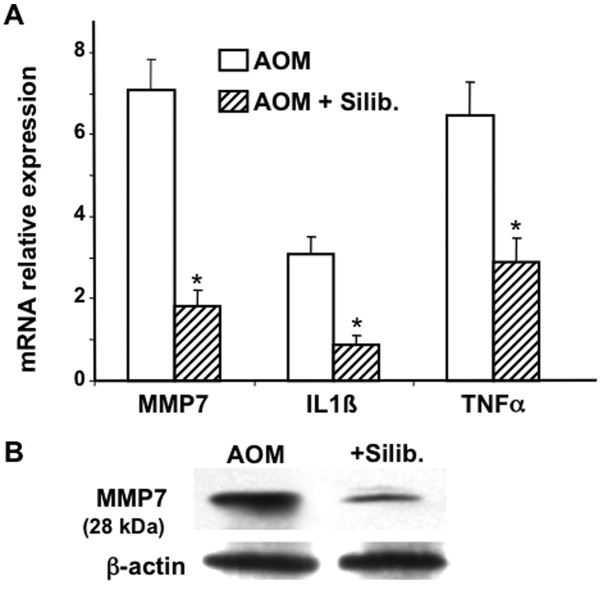
(A) Changes in the mRNA expression levels of pro-inflammatory cytokines (IL1β, TNFα) and matrix metalloproteinase (MMP7) in the colonic mucosa of AOM-injected rats (n=8) and silibinin-treated AOM-injected rats (n=8). Real-time PCR was performed in triplicate wells. The mRNA level from colonic mucosa of saline-injected control rats was used in all determinations as an external reference. The level of β-actin mRNA of each sample was used as an internal reference to normalize the data. The mRNA fold changes (mRNA relative expression) were expressed relative to the corresponding mRNA mean value found in the colonic mucosa of saline injected rats (n=8). Data are presented as the mean ± SE, ^*^P<0.01. (B) Western blot analysis of MMP7 protein expression in the mucosal samples of AOM-injected control rats (AOM) and silibinin-treated (+Silib.) AOM-injected rats; β-actin was used as an internal control.

We examined the differential genetic expression of biomarkers involved in the inflammatory response (MMP7, IL1β, TNFα). Matrix metalloproteinases, such as MMP7, are involved in both early and late processes of carcinogenesis through the degradation of the extracellular matrix and basement membranes (20). We showed here that the amount of MMP7 mRNA was enhanced by 7-fold in the colonic mucosa of AOM-injected rats compared to that of saline-injected rats (Fig. 2A). Silibinin treatment of AOM-injected rats caused a significant downregulation of MMP7 gene expression. In addition, we showed that the amount of MMP7 mRNA was correlated to the amount of MMP7 protein (Fig. 2B). It has been reported that the transcription of the metalloproteinase gene is positively regulated by cytokines and growth factors such as interleukins (IL1β) or TNFα (21) suspected to be associated with the formation of colorectal adenoma in humans (22,23). Accordingly, our data showed the upregulation of both IL1β (3-fold) and TNFα (6-fold) in the colonic mucosa of AOM-injected rats compared to the expression profiles of the same genes in the colonic mucosa of saline-injected rats. Silibinin treatment of AOM-injected rats reduced significantly (P<0.01) the expression of these inflammatory cytokines (Fig. 2A).

### Silibinin activates apoptotic cell death

**Figure 3:**
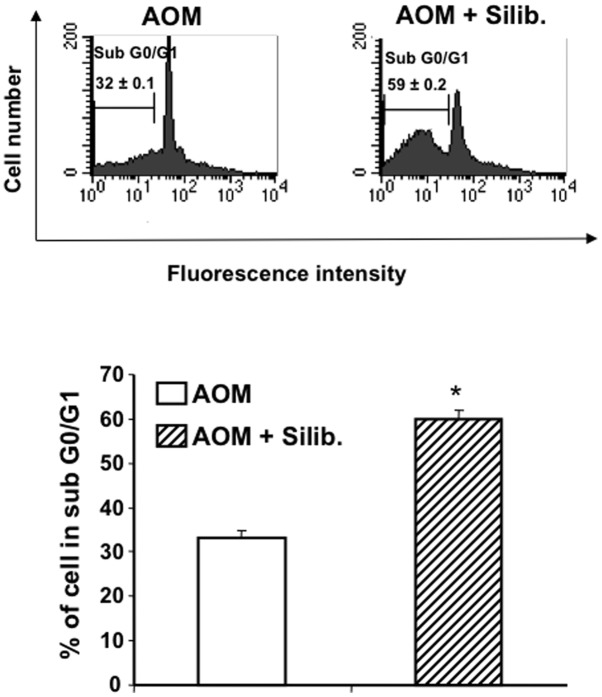
Effects of silibinin on cell death. Mucosal samples were stained with propidium iodide for the measurement of hypodiploid bodies and analyzed by flow cytometry as detailed in the Materials and methods section. The upper panel shows representative FACS histograms. The hypodiploid population is located in the sub G0/G1 region. AOM, AOM-injected controls; AOM + silib., AOM-injected rats treated with silibinin. In the lower panel the percentage of cells in the SubG0/G1 region is indicated. Data are presented as the mean ± SE, ^*^P<0.01.

**Figure 4:**
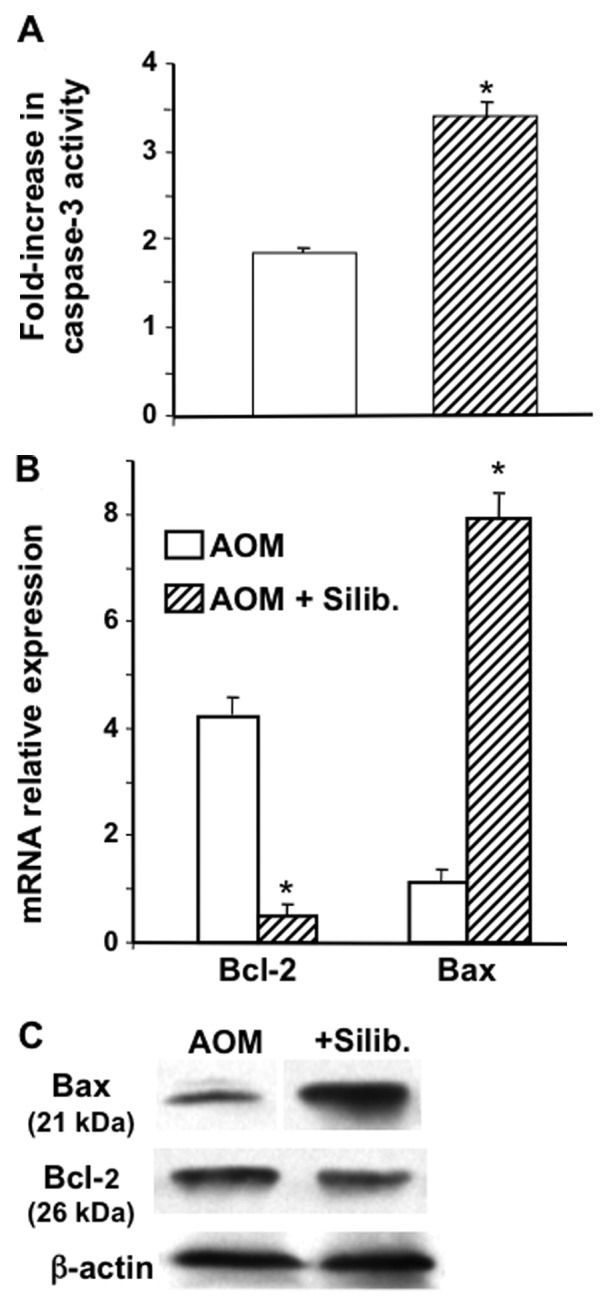
(A) Measure of caspase-3 activity in colonic mucosal samples corresponding to the fold-increase in caspase activity in samples obtained from AOM-injected control rats and AOM-injected rats treated with silibinin relative to the level found in the colonic mucosa of saline-injected control rats. Data are presented as the mean ± SE, ^*^P<0.01. (B) Changes in the mRNA expression levels of Bcl-2 and Bax in the colonic mucosa of AOM-injected rats (n=8) and silibinin treated AOM-injected rats (n=8). Real-time PCR was performed in triplicate wells. The mRNA level from colonic mucosa of saline-injected control rats was used in all determinations as an external reference. The level of β-actin mRNA of each sample was used as an internal reference to normalize the data. The mRNA fold changes (mRNA relative expression) were expressed relative to the corresponding mRNA mean value found in the colonic mucosa of saline injected rats (n=8). Data are presented as the mean ± SE, ^*^P<0.01. (C) Western blot analysis of the pro-apoptotic Bax protein and of the anti-apoptotic Bcl-2 protein expression in the mucosal samples of AOM-injected control rats (AOM) and silibinin-treated AOM-injected rats (+Silib.); β-actin was used as an internal control.

We used flow cytometry in order to assess the amount of dead and dying cells present in the colonic mucosa of AOM-injected rats after 7 weeks of treatment with silibinin. After induction of cell death, DNA is degraded leading to DNA content <2n/cell. These cells are detected by flow cytometric analysis in the subG0/G1 region (24). Our data showed that the subG0/G1 population of colonic mucosal cells was significantly increased after silibinin treatment. The percentage of hypodiploïd cells rose from ∼30% in AOM-injected rats to 60% in AOM-injected rats treated with silibinin (Fig. 3). Furthermore, we found that the enhanced amount of dead cells was correlated with a higher level of caspase-3 activity in the mucosa of silibinin-treated rats (Fig. 4A). These data are in accordance with our previous study showing that silibinin activates caspase-dependent apoptotic signalling pathways in human colon cancer cells (16). In addition, we compared the expression of the anti-apoptotic Bcl-2 and pro-apoptotic Bax mRNA and proteins. We found that the colon of AOM-injected rats exhibited a significant upregulation of the anti-apoptotic Bcl-2 mRNA (4-fold) when compared to saline-injected rats, the amount of Bax mRNA remaining low after AOM injection (Fig. 4B). Silibinin treatment caused a significant drop of Bcl-2 mRNA and protein expression in AOM-injected rats in contrast to Bax transcript and protein, which were significantly upregulated (Fig. 4B and C). Thus silibinin treatment caused a switch in the Bcl-2/Bax ratio, which was elevated (Bcl-2/Bax >1) in the mucosa of AOM-injected rats and was reversed (Bcl-2/Bax <1) after silibinin treatment.

## Discussion

The rodent AOM model of colon carcinogenesis represents a valuable approach for the development of strategies for chemoprevention. This model recapitulates many of the clinical, pathologic and molecular features occurring in sporadic human colorectal cancer (80% of cases), including crypt cell hyperproliferation and ACF formation that are considered as premalignant precursors (7,8). The preneoplastic lesions such as ACF which are detected 30–45 days after AOM administration, have been extensively used as endpoint in short-term chemopreventive studies (25,26). In fact, ACFs are considered to be the ‘gold standard’ of colon carcinogenesis biomarkers (27,28).

In the present study, we show that the flavonolignan silibinin administered daily by intragastric intubation for 7 weeks to rats after AOM injection caused a 50% reduction in the number of preneoplastic lesions (aberrant and hyperproliferative crypts) at the surface of the colon. The dysregulation of crypt cell proliferation in the colon of AOM-injected rats was associated with the activation of several biomarkers of the inflammatory response including MMP7, IL1β and TNFα. Evidence is emerging that members of the MMP family serve as potential markers for early detection and cancer risk assessment. It has been reported that genes of the MMP family such as MMP7 are activated during the early period of ACF formation (29,30). Accordingly, our data indicate that MMP7 gene expression was enhanced in the colonic mucosa of AOM-injected rats, and that the amount of MMP7 mRNA and protein was significantly (P<0.01) reduced by silibinin treatment. Furthermore, we have observed that silibinin drastically reduced IL1β and TNFα mRNA expression. These inflammatory cytokines may play a key role during carcinogenesis and tumor formation as the transgenic overexpression of IL1β in gastric mucosa was sufficient to induce gastric cancer in mice (31).

Progressive inhibition of apoptosis has been described during the transformation of colorectal epithelium to carcinoma (32). Expression of the p53 gene and those of the bcl-2 family (the best known of the genes involved in the regulation of epithelial cell apoptosis) also change progressively as the tissue passes through the consecutive stages of normal mucosa, adenoma and carcinoma (17,33.) While studies had been focused on cancer growth pathways, over the last decade new biological strategies based on the activation of cancer cell apoptosis have been developed in order to reduce mortality of cancer patients (34).

In our previous study, we showed that silibinin was able to activate caspase-dependent apoptotic pathways in human colon adenocarcinoma cells and in their derived metastatic cells (16). Accordingly, we report here that silibinin activated cell death in the colonic mucosal cells of AOM-injected rats and that this effect was correlated with caspase-3 activation. The carcinogenic process requires alterations in the balance between cell renewal and cell death that regulate normal cellular homeostasis in the colonic mucosa. Indeed, we showed in the present report that the AOM-triggered crypt cell hyperproliferation in the colonic mucosa was associated with the upsurge and development of preneoplastic lesions (ACF), but also with an elevated Bcl-2/Bax ratio (ratio >1). Our data showed that silibinin treatment induced the upregulation of the pro-apoptotic Bax protein and gene expression and the reversal of the Bcl-2/Bax ratio (ratio <1).

Silibinin has already been reported to exert potent proapoptotic effects on *in vivo* and *in vitro* cancer models (16,35). Here we show that silibinin-triggered apoptosis may be at least in part responsible for its overall efficacy in inhibiting AOM-induced ACF formation in rat colon. At a molecular level, these effects were associated with silibinin-induced changes in the expression of apoptosis-related genes such as the subsequent Bcl-2/Bax ratio (anti-apoptotic vs. pro-apoptotic gene and protein levels) which can be used as an indicator of chemopreventive efficacy, as previously reported for other drugs (36,37) and as shown here for silibinin.

Herein, we report that intragastric feeding of silibinin to AOM-injected rats exerted various anticarcinogenic and protective effects on the colonic mucosa at early post-initiation stages. Silibinin treatment reduced AOM-induced crypt cell hyperproliferation and ACF formation. At a molecular level, silibinin exhibited multi-target effects on preneoplastic colonic mucosa including the inhibition of pro-inflammatory mediators such as TNFα, IL1β and MMP7, and activation of various pro-apoptotic processes indicating the ability of silibinin to promote normal cellular homeostasis.
